# Supplementary material for: Evaluation of in vitro osteoblast and osteoclast differentiation from stem cell: a systematic review of morphological assays and staining techniques
Source: PeerJ. 2024 Jul 25;12:e17790. doi: 10.7717/peerj.17790 (PMC11283775; doi:10.7717/peerj.17790)
Supplement: Supplemental Information 2 [file peerj-12-17790-s002.docx]

**The rationale for conducting the systematic review and meta-analysis**

The rationale of this review is to understand human stem cell differentiation into osteoblasts and osteoclasts for applications in bone regeneration and diseases. Numerous morphological techniques have been used to assess this osteoblast and osteoclast differentiation, however, a comprehensive review of their application and effectiveness is still lacking.

This systematic review will fill the gap by analysing existing literature and providing insights into different techniques. This review will fill the gap in knowledge by comprehensively analyzing various morphological techniques in evaluating osteoblasts and osteoclast differentiation of human stem cells. With this, this review identifies the most reliable and commonly used osteoblast and osteoclast morphology analysis as guidance for future research. In addition, it also looks at the knowledge lacking for less frequently used techniques and the need to explore novel methods. Therefore, this review offers to address an important gap in cellular morphological knowledge and provide some insight into various techniques for researchers in stem cell differentiation especially in bone development.

**The contribution that the meta-analysis makes to knowledge in light of previously published related reports, including other meta-analyses and systematic reviews.**

The contribution of this study was based on analysing 254 relevant studies systematically based on the morphology of the cells, resulting in the selection of 14 studies for future analysis. The outcome of this identifies trends in morphological techniques application for assessing osteoblast and osteoclast differentiation with the most used techniques being Alizarin Red S and TRAP staining respectively. This review also focuses on the type of stem cells, hence will provide valuable insight for researchers involved in human stem cell bone differentiation.
